# Information Generated by the Moving Pinnae of *Rhinolophus rouxi*: Tuning of the Morphology at Different Harmonics

**DOI:** 10.1371/journal.pone.0020627

**Published:** 2011-06-17

**Authors:** Dieter Vanderelst, Jonas Reijniers, Jan Steckel, Herbert Peremans

**Affiliations:** 1 Department MTT-FTEW, Active Perception Lab, University Antwerp, Antwerp, Belgium; 2 Department of Biology, University Antwerp, Antwerp, Belgium; University of Sussex, United Kingdom

## Abstract

Bats typically emit multi harmonic calls. Their head morphology shapes the emission and hearing sound fields as a function of frequency. Therefore, the sound fields are markedly different for the various harmonics. As the sound field provides bats with all necessary cues to locate objects in space, different harmonics might provide them with variable amounts of information about the location of objects. Also, the ability to locate objects in different parts of the frontal hemisphere might vary across harmonics. This paper evaluates this hypothesis in *R. rouxi*, using an information theoretic framework. We estimate the reflector position information transfer in the echolocation system of *R. rouxi* as a function of frequency. This analysis shows that localization performance reaches a global minimum and a global maximum at the two most energetic frequency components of *R. rouxi* 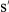 call indicating tuning of morphology and harmonic structure. Using the fundamental the bat is able to locate objects in a large portion of the frontal hemisphere. In contrast, using the 1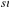 overtone, it can only locate objects, albeit with a slightly higher accuracy, in a small portion of the frontal hemisphere by reducing sensitivity to echoes from outside this region of interest. Hence, different harmonic components provide the bat either with a wide view or a focused view of its environment. We propose these findings can be interpreted in the context of the foraging behaviour of *R. rouxi*, i.e., hunting in cluttered environments. Indeed, the focused view provided by the 1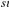 overtone suggests that at this frequency its morphology is tuned for clutter rejection and accurate localization in a small region of interest while the finding that overall localization performance is best at the fundamental indicates that the morphology is simultaneously tuned to optimize overall localization performance at this frequency.

## Introduction

Irrespective of the species, echolocating bats usually emit ultrasonic calls consisting of different harmonics (see [1], [2] for examples). Indeed, harmonicity is something really hard to avoid; all mammals generate harmonics of the fundamental frequency with their vocal folds. Nevertheless, bats seem to have at least some control as to which of their harmonics to emit at each call. They can also control the relative strength of their harmonics. In the species studied thus far, both the emission pattern and the directional hearing sensitivity vary considerably as a function of frequency [3]–[6] i.e. the way the head morphology of bats shapes the outgoing and the incoming sound field changes radically with frequency. For example, the direction in which most energy is emitted by noseleaved bats is different for their various harmonics [3]. As the sound fields provide bats with the cues to locate echoes in space, this suggests that the different harmonics in their calls provide bats with different amounts of localization information. Also, localization performance might be different for different parts of the frontal hemisphere. In addition, the morphology of the pinnae and the face might be shaped in order for the bat to be able to gather information from different regions if required by the task.

**Figure 1:**
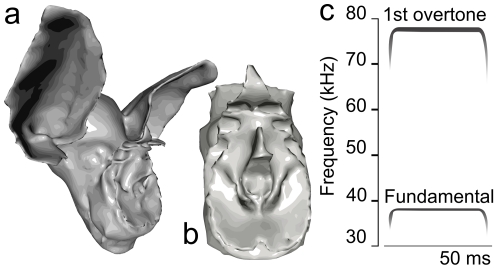
3D models of *R. rouxi* and spectrogram. (a) Rendering of the *R. rouxi* model used in this study. (b) Model of the noseleaf used. (c) Schematic spectrogram of a *R. rouxi* call.

In this paper, we test the hypothesis that the morphology of bats has been shaped such that the different harmonics in their calls allow the bat to localize targets in different region of the frontal hemisphere with varying precision. To this end, we employ the recently developed information theoretic framework we proposed to quantify the localization performance in bat echolocation systems [7]. We selected *Rhinolophus rouxi* to test our hypothesis since the family of Rhinolophidae emit pulses consisting of a set of narrowband harmonics [1], [8], [9]. In particular, *R. rouxi* uses pulses with a strong 1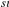 overtone of about 80 kHz and a weaker fundamental of about 40 kHz (see spectrogram figure 1).

The fact that *R. rouxi* uses pulses consisting of one or two narrowband components allows us to operationalize our hypothesis by calculating the localization performance at a range of frequencies that hypothetically could be used by the bat. This allows us to tell whether (1) the morphology optimizes the localization performance for the frequencies (harmonics) actually used by the bat and (2) in what way localization performance differs for the two harmonics.

While the calls of Rhinolophidae are often preceded by a short upward sweep and/or followed by a short downward sweep, we only consider the constant frequency (CF) component of the calls of *R. rouxi* in our analysis. The limited bandwidth and relatively small energy in the frequency modulated (FM) component of their call has been taken to indicate that Rhinolophidae rely less on the spectral cues that are used for echolocation by bats emitting broadband calls [10]–[13]. Rhinolophidae are known to hawk aerial prey. In addition, they also hunt from perches from which they perform very short foraging flights (less than 1 second) ambushing passing prey [8], [9], [14], [15]. *R. rouxi* has been observed to omit the FM component in 90 percent of its calls while hanging from a perch and scanning the surroundings for flying insect prey [8], [9]. Moreover, some species have been observed to emit no FM components at all. This strongly suggests that CF bats do not rely on spectral cues while locating prey from a perch. Indeed, it has been suggested that the FM component of the call is used to gauge the distance to targets ([16] and see [8], [9] for references). To compensate for the lack of spectral cues, it is hypothesized that CF bats employ behavioral strategies that most likely generate cues to perform localization using the CF component of their calls [5], [17], [18].

While perching, these animals move their pinnae vigorously when emitting echolocation calls [19]–[21]. While the right ear is moving forward, the left moves backward. The tips of ears move through an arc of about 30 degrees or 1 cm [21]. The pinnae movements are synchronized to the time of arrival of echoes. This is, the ears are at their extreme positions between the reception of echoes and sweep to the other extreme position during the reception of an echo [19], [21]. Ear movements are exhibited while hanging from a perch as well as during flight (see ref [19] and a Nature video associated with ref. [22] shows a rhinolophid bat moving its ears during flight). As ear movements are a critical aspect of the echolocation behavior of *R. rouxi* we take them into account in our model.

### Model Description

**Figure 2:**
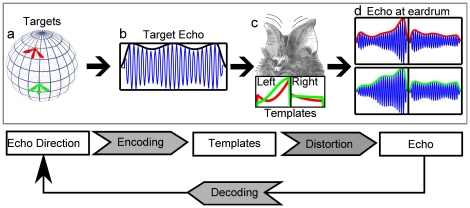
Illustration of the classification model used to evaluate the localization performance of *R. rouxi*. Top row: (a) Targets at different locations (red and green insect) yield a similar echo (b) of which the amplitude is modulated due to movements of the targets. The movement of the pinnae (c) during the reception of the echo modulates the amplitude of the echo (d). This modulation depends on how the pinna movement moves the target through the HRTF of *R. rouxi* (illustrated in c bottom). (d) The final amplitude modulated echoes for the read and the green target at both tympanic membranes. Bottom Row: An algorithmic explanation of the model. The direction from which an echo originates is encoded by the amplitude modulation introduced by the ears (templates). Distortions of this encoding occur due to amplitude modulations introduced by the moving targets. The bat tries to decode the direction of the echo. Our model estimates the mutual information between the echo and the echo direction.

To evaluate the contributions of the harmonics in the calls of *R. rouxi* we employ an information theoretic model of the echolocation task as illustrated in figure 2. The basic assumption of this model is that localization of a target can be considered as a template matching task [7], [23], [24].

A target, e.g., a fluttering insect (figure 2a), produces an echo containing typical target-induced modulations (figure 2b) that are picked up by the bat's moving external ears (figure 2c). We assume, that the pinnae move either up or down during the reception of the echo [19]–[21]. Ear movement introduces additional amplitude modulations of the echo at both tympanic membranes (figure 2d). The exact way in which the echo is modulated depends on the augmented head related transfer function (AHRTF), i.e., the combination of the emission directivity and the head related transfer function, of the bat. Each different azimuth-elevation position of a target with respect to the bat corresponds to a different expected modulation pattern at the left and the right ear (illustrated in figure 2c). These expected modulation patterns are termed templates in the remainder of the paper. As the modulation of an echo by the moving pinnae depends on the position of a target, the modulation encodes the position of the target. In figure 2, the red and the green target are positioned at different locations with respect to the bat. Therefore, the modulation of the echo by the bat's ear movements is different for both insects as illustrated in figure 2c–d.

We assume that *R. rouxi* uses the amplitude modulation of the echo due to the pinna movement to estimate the azimuth and elevation of the target. Most studies of echolocation in Rhinolophidae concern frequency modulations introduced into the echo by targets (glints) that allow the bat to identify prey and separate the target from the background (e.g. [25]). In this study, we do not assume that these frequency modulations are of importance for determining the azimuth and elevation of the target. Moreover, it has been suggested that Rhinolophidae might introduce frequency modulations onto the returning echoes by moving their pinnae due to Doppler shifts thereby creating cues from which to infer azimuth and elevation [26]. However, simulations strongly suggest that this mechanism does not provide stable enough cues to estimate the azimuth and elevation location of targets (see [27] for an evaluation of this hypothesis).

If no noise sources would be present, comparing the amplitude modulation in strong echoes from static reflectors with the expected modulations corresponding to each azimuth-elevation position and taking the best matching template as an estimate of the direction of the echo would yield perfect localization performance. However, in reality several factors impose a limit on the localization performance. First, the bat has to classify the echo in the face of unknown modulation imposed on the echo by the fluttering of the target (for this reason the echo illustrated in figure 2b is modulated). In addition, the localization performance of the bat will be limited by the intensity of the echoes. The amplitude of a weak echo can only be weakly modulated as amplitude modulations that reduce the amplitude of the echo below the detection threshold of the bat will be effectively truncated. The detection threshold of the bat is determined by the internal and environmental noise. A final factor interfering with the matching between measurements and templates is uncertainty about the strength of the echo. The strength of the echo, before filtering by the pinnae, is unknown to the bat. The bat might confuse a strong echo that originated from a strong reflector with that from a weak reflector in a direction for which its sonar system is highly sensitive. In sum, three factors limit the matching between stored templates and measured amplitude modulations: amplitude modulations generated by the target, the signal to noise ratio and uncertainty about the echo intensity before filtering by the pinnae. The information theoretic model of the echolocation task we use in this paper takes into account these three factors.

Our model employs Bayes' theorem to calculate the uncertainty in matching measurements to templates in the face of the noise factors described. We express this uncertainty in Shannon entropy as a number of bits (see [7]). In particular, we calculate, for measurements originating from each azimuth-elevation position, how likely it is the bat will assign the measurement to each and every azimuth-elevation position. This uncertainty about the true target position is expressed in bits. The uncertainty about the true origin of a measurement depends on the templates employed (i.e., the amount of information missing to uniquely specify the target's position). Some template sets will encode the origin of the echo with less ambiguity than others in the presence of the distortions introduced by the echo reflection process. As the HRTF of *R. rouxi* is different for each frequency, different frequencies yield different template sets. In this paper, we compare the average entropy about the true origin of a measurement for templates based on different frequencies. As the AHRTF (and thus the template set) is determined by the morphology of the bat, we can test whether the bat's morphology is tuned to a particular frequency by evaluating whether the template set for this frequency yields better performance than those based on other frequencies.

Entropy has proven a powerful measure to quantify the performance of sensory systems under different conditions [28]–[30]. Moreover, the model can be considered as an adaptation and extension of the models used in cognitive psychology to model human categorization performance, e.g. [31].

While the hearing directionality of *R. rouxi* has been measured [5], this is not the case for the emission directivity. However, simulation methods have become available that allow the evaluation of the spatial sensitivity of the echolocation system of bats at a high resolution [3], [6], [32]–[34]. Among these simulation methods, Boundary Element Methods (BEM) are well suited to simulate both the emission and hearing directionality of bats [3], [6]. Furthermore, BEM is thus far the only simulation method that has been formally validated for the simulation of HRTFs of small mammals (for the bat *Phyllostomus discolor* [6] and for gerbils [35]). Using BEM to simulate the spatial sensitivity of a bat requires a 3D model of the morphology of the head of the species under study. In our lab, we have developed a method to create such a model from CT data [6]. The 3D model of *R. rouxi* used in this study is rendered in figure 1a–b.

By using a model of the echolocation task and simulated template sets it is possible to evaluate the performance of the bat's morphology in encoding the azimuth and elevation positions of targets at different frequencies. Indeed, this is impossible to test in an experiment as this would require the bat to shift its dominant frequency over a large range. Moreover, even if the bat could be induced to shift its frequency range, the specialization of its cochlea and neural apparatus would introduce a confounding factor making it impossible to evaluate the contribution of the morphology to the localization performance.

Rhinolophidae have a very baroque facial morphology (see fig. 1). They are characterized by large noseleaves with a number of furrows. To directly test for the contribution of this facial morphology to the localization performance, we ran a simulation in which we substituted the emission pattern of *R. rouxi* by that of two omni-directional emitters spaced 4.2 mm apart (i.e. half the wavelength at 80 kHz). This simulation omits any effect of the facial features of *R. rouxi* save for the spacing of its nostrils.

## Results

The performance of the model in matching templates and measurements critically depends on the assumed echo strength or signal to noise ratio of the echo. In the lab, fixated *R. rouxi* were found to call with an amplitude of about 105 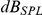 (at 10 cm in front of the bat) [36]. *R. rouxi* hunts mostly for insects with a wing length smaller than 10 mm [37]. Fluttering insects of this size return an echo that is up to 50–60 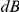 weaker than the impinging sound (depending on the frequencies used) [38]. In other words, as little as 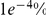 of the impinging energy might be reflected in the direction of the emitter. Therefore, we evaluated the performance of the model for echoes ranging from 0 to 50 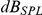 in steps of 5 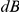 as this contains all echo strengths *R. rouxi* is likely to encounter. Moreover, we evaluate the performance of the model for three values of the amplitude modulation of the echo introduced by the fluttering of the target referred to as Low, Medium and High noise levels. The exact meaning of these values is explained in the methods section below.

### The head related transfer function

**Figure 3:**
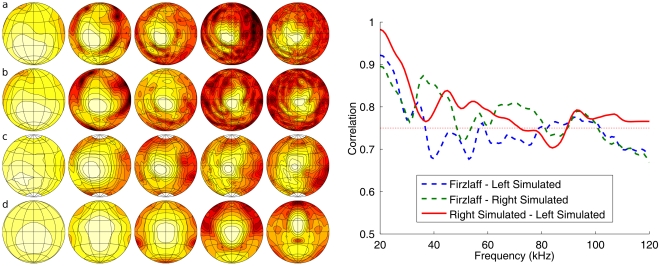
Simulated HRTF and emission patterns. Left: The simulated HRTF and emission pattern (frontal hemisphere only). Row a: simulated spatial sensitivity of the left ear (mirrored). Row b: simulated spatial sensitivity of the right ear. Row c: spatial sensitivity of the right ear of a *R. rouxi* specimen measured by Firzlaff and Schuller [5]. Row d: the simulated emission pattern. All plots in 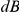 and normalized such that the maximum is 0. Contour lines are 3 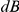 apart. The columns depict different frequencies: 20, 40, 60, 80 and 100 kHz. Right: The correlations of the simulated hearing sensitivity of the left and the right ear with the sensitivity measured by Firzlaff and Schuller [5] as a function of frequency. The red horizontal line denotes 0.75. This is the average between-specimen correlation found in ref. [6].

The HRTF of *R. rouxi* has been measured by Firzlaff and Schuller [5]. We compare the simulated HRTF with the data collected by Firzlaff and Schuller [5] (figure 3). The spatial sensitivity simulated for the left and the right ear of our specimen of *R. rouxi* matches well with the measured data. The correlation between the simulated and the measured spatial sensitivity is consistently larger than 0.5 (see figure 3). When comparing measurements of the spatial sensitivity of different specimens of *P. discolor*, correlations also varied between 0.9 and 0.5 (average correlation: 0.75. This value is indicated by a red line in the right panel figure 3) [6]. Therefore, mismatches between the simulated and the measured HRTF are not larger than individual variations within a species.

Finally, it is important to note that the difference between the simulation of left and the right ear were about the same than between the left ear and the measurements collected by Firzlaff and Schuller [5]. This indicates that deviations between the measurements and the simulations are not larger than the within specimen variation.

### Spatial distribution of entropy

**Figure 4:**
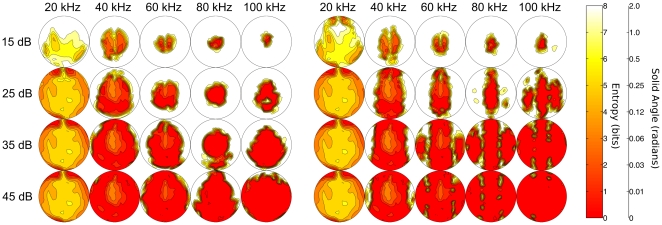
Spatial distribution of localization entropy. The entropy (in bits) about the origin of an echo as a function of reflector position, signal to noise ratio (15 to 45 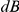) and frequency. Left: results for the model in which the actual facial morphology of *R. rouxi* was included. Right: results for a model in which the emission pattern of *R. rouxi* was replaced by that of two isotropic sources. The plots cover the frontal hemisphere of the bat (−90 to +90 degrees in azimuth and elevation).

The entropy about the origin of an echo, as expressed in bits of information that remain to be specified to know the target position exactly, varies considerably across the frontal hemisphere (figure 4). The part of the frontal hemisphere in which *R. rouxi* is predicted to be best at locating incoming echoes depends on the frequency and the strength of the echo. For weak echoes (15 & 25 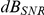), the model performs best in a central part of the hemisphere. This region in which the entropy about the origin of the echo is the smallest decreases in size and shifts to slightly higher elevations as frequency increases. The area in which localization is possible is the smallest around 80 kHz. Echoes with a strength in the range of 5 to 20 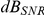 can not be located if they originate from the peripheral region. The entropy about the origin of echoes is about 8 bits in this region for all frequencies considered. This is the chance level performance, i.e., an entropy of 8 bits means that all target locations are equally likely in the numerical experiments conducted here.

As echoes become stronger, the overall performance of the model increases. However, the best localization performance is no longer strictly obtained in a central region. Indeed, for frequencies around 40–60 kHz, performance is better in the periphery. The reason for this, can be seen by comparing the performance for 35 and 50 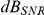 for all frequencies. Increasing the strength of the echo saturates the performance of the model at 35 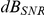 in the central region. Even at high frequencies, the performance of the model does not increase any further for echoes stronger than about 30 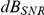. In contrast, in the periphery, *R. rouxi* can exploit an increase in echo strength up to about 50 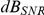. It is noteworthy that the performance at low frequencies (below 30 kHz) is bad even for very high echo strengths. In sum, the simulation reveals a complex three-way interaction between the origin of an echo, the strength of the echo and the echo's dominant frequency determining the entropy about its origin.

The behavior of the model in which the facial morphology was replaced by two isotropic sound sources is similar to that of the original model. The main difference between the results of both models is the increased performance of the model with the isotropic sources. The region in which the predicted localization performance is high, is systematically larger when the facial morphology is omitted from the simulation.

### Entropy as a function of frequency

**Figure 5:**
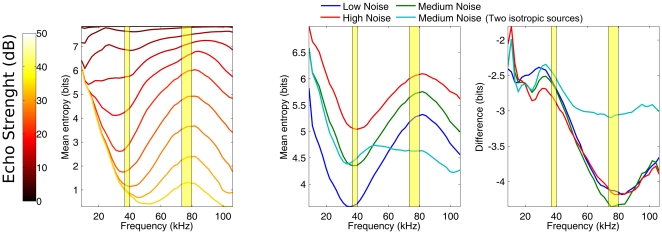
Localization performance as a function frequency. Higher values denote higher entropy and lower localization performance. (a) The performance of the model for different signal to noise ratios as a function of frequency for a medium noise level averaged across the frontal hemisphere (see Methods section). (b) The performance as a function of frequency averaged across the signal to noise ratios for three levels of noise and a model in which the emission pattern was replaced by that of two isotropic sound sources. (c) The difference between the azimuth-elevation position for which performance is the worst and the best as function of frequency. This plot shows that, at around 80 kHz, the range in localization performance across the frontal hemisphere is the largest. Moreover, the range in performance across locations in the frontal hemisphere is reduced by replacing the emission pattern of *R. rouxi* by that of two isotropic sources. The yellow regions are 95% confidence ranges for the fundamental and 1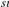 overtone in the call of *R. rouxi* as estimated from the data provided in ref. [8].

**Figure 6:**
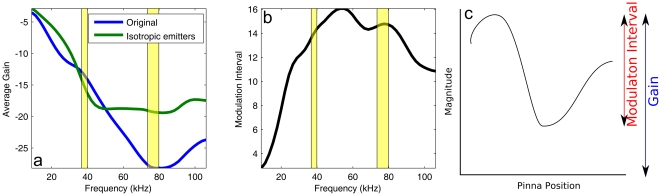
Changes in template properties as a function of frequency. (a &b) Gain and modulation interval of the templates as a function of frequency. The range in 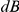 of a template is defined as the difference between the highest and the lowest gain (in 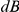) in the template. The yellow regions are 95% confidence ranges for the fundamental and 1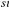 overtone in the call of *R. rouxi* as estimated from ref. [8]. In plot (a), different lines are drawn for a model in which the original facial morphology was used and one in which the facial morphology was replaced by two isotropic sources. Note that for the modulation interval for both models is the same as the modulations are introduced by the moving ears only. Therefore, plot (b) contains only a single line. (c) This plot explains the terms ‘gain’ and ‘modulation interval’ plotted in (a). The black line depicts a stylized template while the colored lines indicate the two terms.

In figure 5a, the average performance of the model in the frontal hemisphere is plotted as a function of frequency and echo strength. The curves tend to show a minimum around 40 kHz (i.e. at the fundamental) while they reach a maximum around 80 kHz (i.e. at the 1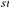 overtone). Indeed, when averaging across echo strengths (figure 5b), it can be seen that the model performs best slightly below 40 kHz and worst slightly below 80 kHz. The reason for the good performance at 40 kHz and the bad performance at 80 kHz can be understood by looking at the properties of the templates at these frequencies (figure 6).

The average gain of the templates reaches a minimum just above 80 kHz. As the templates were normalized per frequency such that the highest gain across all templates is zero 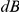, this indicates that the energy in the templates around 80 kHz is more (as compared to other frequencies) directed towards the center at the cost of the peripheral templates. Indeed, the normalization allows the average gain to be interpreted as the inverse of the directivity index. The lower average gain for peripheral templates explains why at this frequency range, localization performance is lowest.

The best performance for frequencies around 40 kHz is explained by a more subtle trade-off between the modulation interval of the templates and their gain.

As can be seen in figure 6, at 40 kHz the slopes of the gain and modulation as a function of frequency are maximal, but have opposite signs. Below 60 kHz, the average within-template modulation interval reduces quickly with lower frequencies. A decrease in the within-template modulation interval decreases the classification performance as this makes discriminating between templates more difficult. On the other hand, decreasing frequency increases the gain of templates, yielding better performance. It seems that around 40 kHz two effects are balanced: the templates have both high enough gains and within-template modulation to enable good localization. It should be noted here, that the within-template modulation interval is strictly due to the movement of the pinnae.

The results of the simulation in which the facial morphology has been replaced by two isotropic sources are also plotted in figure 5b–c. By omitting the facial morphology, the performance of the model increases around 80 kHz as the focusing of the energy is reduced (figure 6a). This confirms the differences in localization performance are mostly due to the redistribution the emitted energy by the facial morphology of *R. rouxi*.

In sum, the facial morphology of *R. rouxi* focuses the emitted energy most around 80 kHz. This reduces the localization of peripheral echoes to such an extent that localization performance across the frontal hemisphere is worse than at any other frequency. In contrast, at around 40 kHz, the emitted energy is spread more evenly across the frontal hemisphere. Moreover, at this frequency, the external ears introduce ample gain variation in the templates. Consequently, the localization performance is best around this frequency.

### Simulation of perch hunting

The simulation results presented in figures 4 and 5 give the expected localization performance as a function of frequency and the strength of the echo. However, the distribution of the strengths of the echoes *R. rouxi* encounters depends on the distances of the passing insects it attempts to capture. Indeed, *R. rouxi* seems to leave its perch only if an insect is close enough to be caught within 0.5 to 1 second after take-off and it restricts its hunting flights to about 5 meters, including the pursuit of insects [8], [9]. The relationship between the maximum distance of interest to the bat and the distribution of resulting echo strengths is complex as it is determined by spherical spreading, atmospheric attenuation and the distribution of the distances between prey and bat.

We use a Monte Carlo technique to simulate the effect of the extent of the foraging patch of *R. rouxi* on the expected echo amplitudes. We run separate Monte Carlo simulations for each frequency. In these simulations we assume foraging extents from 1 to 5 meter (in steps of 1 m). Furthermore, we assume that *R. rouxi* hunts for insects returning an echo 40 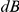 weaker than the impinging sound while hunting from a perch [37], [38]. This is, we assume that 0.01% of the energy is reflected back to the emitter from the insect. Based on the measurements performed by Firzlaff and Schuller [5] on the maximum gain of the external ears of *R. rouxi*, we set the maximum gain of the external ears to 12 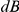. Finally, we assume *R. rouxi* emits its call with an amplitude of 105 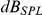 [36].

For each assumed foraging distance, we generated 1000 random locations for the prey within a radius given by the current range centered around the bat. Next, based on the 1000 distances between the bat and the prey, we calculated the strength of each of the echoes received by the bat based on the spherical spreading, atmospheric attenuation [39], [40], ear gain and reflector strength. For each amplitude, the expected localization performance of the model was retrieved (as plotted in figure 5a for Medium Noise Level). The localization performance was then averaged across the 1000 replications.

**Figure 7:**
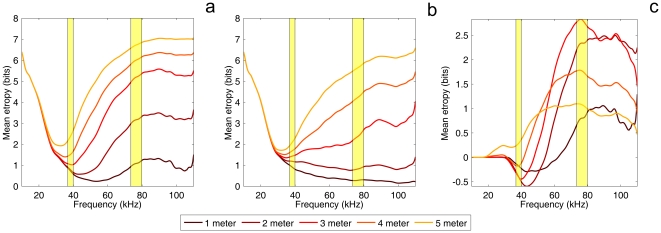
Results of the Monte Carlo simulation of perch hunting. (a) The average entropy about the location of an insect as function of the used echolocation frequency and the distance between the bat and the insect (which determines the echo strength) as established by a Monte Carlo simulation. (b) Similar as (a) but for the model in which the emission pattern of *R. rouxi* was replaced by that of two isotropic sources. (c) Difference between (a) and (b). Note that the scale of the y-axis is different in (c). The yellow regions are 95% confidence ranges for the fundamental and 1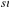 overtone in the call of *R. rouxi* as estimated from ref. [8].

Figure 7a, shows that when including the parameters known about the behavior of *R. rouxi*, the tuning of the echolocation system for frequencies little below 40 kHz is confirmed. The curves in this figure reach a minimum around the fundamental. Moreover, the lack in performance around 80 kHz is also shown as the performance reaches a lower plateau around this frequency. Indeed, performance reaches a global minimum around 80 kHz and performance does not increases for higher frequencies. It is the atmospheric attenuation for higher frequencies that prevents performance to increase for frequencies above 80 kHz (as was the case in figure 5 were atmospheric attenuation was not taken into account).

Figure 7b plots the simulated performance for the model in which the facial morphology was replaced by two isotropic emitters. When comparing these results with those for the original model (difference plotted in fig. 7c) it becomes clear that the model using two isotropic sources outperforms the original model. In addition, localization performance no longer reaches a global minimum at 80 kHz.

The largest advantage of this model over the original model can be noted around 80 kHz and above. The difference between the model with and without facial morphology is largest for foraging ranges of 3 meter and when using 80 kHz. In addition, the frequency at which performance is best shifts from 40 to 30 kHz as the assumed hunting range is extended beyond 3 meter. This is due to atmospheric absorption becoming a more important factor at these ranges. This also points to the morphology not only being tuned for certain frequency ranges but also for a foraging range of about 3 meter.

## Discussion

In this paper we investigated whether the morphology of bat might be shaped such that different harmonics provide different views of the world. We used *R. rouxi* as a test case. Grinnell and Schnitzler [41] have measured the emission pattern of *R. ferrumequinum*. However, they could not evaluate how the emission pattern would change as a function of frequency. The simulation method presented here allows to calculate the outgoing and the incoming sound field at any frequency [3], [6].

From the simulations, it follows that the main lobe is smallest at the center frequency of *R. rouxi*. Our model of the information transfer in an echolocation task predicts that having the highest directionality at the dominant frequency has an important functional consequence for *R. rouxi*. Indeed, at this frequency its predicted localization performance of peripheral targets is worse than for any frequency evaluated in this paper. This is because at 80 kHz the gain of the templates declines most rapidly for more peripheral target positions (see figure 6a). While hindering the ability to localize peripheral targets, this will decrease the influence of clutter echoes from these positions. In the mathematical model employed here (see Methods), clutter echoes would result in a larger values of 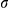 and a lower signal to noise ratio, both of which will decrease performance (see also ref. [3] for simulations on the effect of clutter echoes in FM bats). Indeed, As 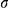 is used to model the noise due to the amplitude modulations introduced by the fluttering target, any other source that introduces amplitude modulations will results in a larger value for 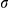. Echoes from clutter will interfere with the target echo and introduce unwanted amplitude variations.

The best overall localization performance is reached around 40 kHz (i.e. the fundamental frequency). The best performance of the echolocation system of *R. rouxi* at its fundamental is due to a trade-off between gain and variation in the template set (see figure 6). Localization performance is enhanced if the pinnae introduce more variation in the templates. Indeed, if ear movements modulate the incoming echo more, estimating the direction of the echo will be easier as the modulations will be more robust against unknown reflector modulations. However, modulating the amplitude of an echo reduces its average gain making the signal more likely to fall below the noise level. Therefore, any echolocation system is faced with a trade-off between introducing more pronounced cues in the echoes and keeping the average strength of the echo as high as possible [7]. From the analysis presented here, it follows that around 40 kHz the morphology of *R. rouxi* strikes the best balance between these conflicting demands.

Currently, it is unclear what *R. rouxi* uses its fundamental for (see also ref. [8] for a discussion). These bats regularly omit the fundamental from their pulses [8], [15]. Furthermore, audiograms based on behavioral measurements and on otoacoustic emissions indicate *R. rouxi* is insensitive to the frequencies in its fundamental [42], [43]. However, neural populations tuned to the frequencies in the fundamental have been found in the superior colliculus and other places in the neural pathway [44]. Moreover, the external ears of *R. rouxi* are most sensitive at the fundamental [5] and occasionally the fundamental is emitted at the same loudness as the 1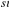 overtone [8]. Our findings suggest that *R. rouxi* has an echolocation apparatus that can provide the bat simultaneously with a focused view (using the 1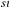 overtone) and a wide view (using the fundamental).

The functional relevance of having an echolocation system with a focused and a wide view modus can be readily inferred when taking into account the ecological background of *R. roux*. Bats of the family Rhinolophidaee are known to hunt in densely cluttered environments [1], [8], [9]. Indeed, the use of CF pulses by Rhinolophidae has been interpreted as an adaption to hunting in cluttered environments [1], [38]. First, clutter is rejected as fluttering and moving targets induce Doppler shifts to which the cochlea is highly sensitive [42], [43]. Second, the high frequency echoes from objects behind a target are highly attenuated because of the atmospheric absorption at these frequencies [38]. Having an echolocation system that is optimized for focusing on a small portion of the world could represent another adaption to the cluttered environment in which it hunts. By emitting 40 kHz pulses it could acquire a general impression of the environment including parameters such a density of the clutter, level of confinement and nearness of large reflectors. Simultaneously, by using 80 kHz it could gather information about the precise location of a specific target in its region of interest with minimal interference from clutter.

The difference in performance for the two harmonics is mostly due to the facial morphology of *R. rouxi*. Replacing the emission pattern with that of two isotropic sound sources reduces the effect of frequency on performance. This suggests that the facial morphology of *R. rouxi* is evolutionary tuned to provide the bat with a focused view at the 1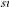 overtone and a wide view at the fundamental. Indeed, in a Monte Carlo simulation of the perch hunting behavior of *R. rouxi* we found that the largest difference between the model with and without the facial morphology at 80 kHz and for a foraging range of 3 meter. This indicates that the facial morphology is not only tuned to a certain frequency but that its effect might also be tuned to a certain foraging range. In addition, in this simulation, the frequency at which performance is best increases from 40 to 30 kHz as the assumed foraging range is extended beyond 3 meter. This also points to the morphology not only being tuned for certain frequency ranges but also for a foraging range of about 3 meter.

Interestingly, our findings, based here on *R. rouxi*, can probably be extended to other members of the same family. The body size of Rhinolophidae is correlated with their dominant frequency [45]. More importantly, the width of the noseleaf is strongly correlated to the resting frequency [46]. Bigger bats (with larger noseleaves) have lower dominant frequencies. This suggests that the clutter rejection mechanism found in *R. rouxi* might also be present in other bats from the same family with a similar call design and ecological niche. Irrespective of their size, they might all make use of the 1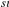 overtone that rejects clutter by focusing on a small frontal region of interest.

The finding that the morphology of *R. rouxi* helps it to reject echoes from peripheral reflectors fits with our recent proposal about the functional role of the noseleaves of FM bats. Recently, we have argued that the noseleaves of bats hunting in cluttered environments are especially suited to reject clutter. Indeed, the functionality of the noseleaf of *Micronycteris microtis*, an FM bat hunting among vegetation, has been interpreted as serving to reject clutter echoes by reducing the degree to which peripheral objects are ensonified. In turn, this reduces the interference between echoes from targets and echoes from spurious objects [3]. See ref. [7] for an information theoretic analysis of the echolocation system of *Micronicteris microtis*.

It is important to note that our analysis does not take into account any processing of the echo in the cochlea of *R. rouxi*. The specialized physiology of the cochlea is not taken into account because we set out to evaluate the influence of the morphology of *R. rouxi* on its localization performance. Therefore, our information estimates should be considered as an upper limit of the actual information transfer. Processing in the cochlea and the auditory pathway can only reduce the information transferred. The low amount of information transferred about peripheral objects at the dominant frequency of *R. rouxi* can not be reversed by neural processing. Further work could aim at estimating the information transfer rate in the cochlea given the specialized functionality of this organ in CF bats. We have previously presented such an analysis for FM bats [30].

Further research could establish whether other species of bats derive different types of information from the various harmonics in their calls. More specifically, our analysis could be extended to bats using frequency modulated calls. Indeed, many of these bats not only change the relative amplitude of the harmonics in their calls. They also can alter the time-frequency structure of their calls (e.g. [47]). The framework and analysis presented in this paper could be used to investigate (and quantify) the functional relevance of this plasticity in the design of the calls.

## Methods

### Simulation of the hearing sensitivity and emission beam

We have reported in detail on the method we use to simulate the directionality of bat echolocation systems and its validation elsewhere [3], [6]. Therefore, we will only report briefly on the simulation methods here. Using BEM to simulate the sound field around an object requires the construction of a detailed mesh model of the object under study. Therefore, the head of a single specimen of *R. rouxi* was scanned with a MicroCT machine using a resolution of 70 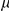
m. After reconstruction of the shadow images, an initial mesh model is obtained using a set of standard biomedical imaging tools. Current computational facilities allow to simulate models containing up to 32,000 triangles. The noseleaf of *R. rouxi* is a very complex structure consisting of two rows of furrows. To construct a highly detailed model of this structure, it was decided to make a separate model of the noseleaf. Therefore, in this paper we use a detailed model of the noseleaf to simulate the emission directionality of *R. rouxi* and a model of the complete head (including a simpler noseleaf model) to simulate the hearing directionality. Both initial models were subjected to several rounds of smoothing and remeshing to reduce the number of triangles in the models to little under 32,000. The maximum edge length of the final head model was 0.5 mm. For the noseleaf model a maximum edge length of 0.35 mm is used. At 110 kHz, the highest frequency employed in the presented simulations, an edge length of 0.5 mm results in a sampling of more than 4 nodes per wavelength which is sufficient to obtain stable simulation results. Figure 1, shows the bat models used in this study together with a spectrogram of *R. rouxi* calls. Five virtual receivers are placed in both the left and the right ear canal of the head model at the approximate position of the eardrum. We report on the average sound field as picked up by both groups of receivers. Furthermore, to simulate the emission beam pattern we placed virtual receivers in both nostrils of the noseleaf model. To obtain the emission beam pattern the complex sound field of the left and the right nostril are summed and we report on the magnitude only. Placing receivers in the noseleaf model to simulate the emission beam pattern is warranted by the reciprocity principle [48] and enhances numerical stability of the simulations [49]. Virtual omnidirectional sources were placed on an imaginary sphere with a diameter of 1 m around the bat head or noseleaf model. The sources were spaced 2.5 degrees apart covering −90 to 90 degrees in both azimuth and elevation (i.e. 10,658 sources). Placing the sources in this regular configuration allows for easy preprocessing of the data. However, this configuration does not uniformly sample the sound field on the sphere. Therefore, we resampled the sound field at equally spaced positions during the processing of the data using the Recursive Zonal Equal Area Sphere Partitioning Toolbox [50]. We assume that all the emitted sound energy stays within the frontal hemisphere i.e., negligible amounts of energy are radiated backward, requiring the normalization of the emission beam patterns of the bats per frequency 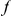


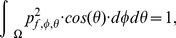
(1)with 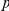 denoting the magnitude of the emission strength for frequency 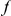 in direction (azimuth  =  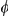, elevation  =  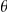) and 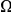 the frontal hemisphere. The spatial sensitivity of the complete sonar system is calculated by pointwise multiplication of the values at corresponding directions for the HRTF and the emission beam pattern at frequency 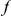 [13]. We assume that ear movements result in rigid rotations of the hearing directionality. In cats it has been shown that this is a good approximation [51]. Furthermore, the ear movements modeled in this paper are modest and can probably be well approximated by rigid rotations of the hearing directionality. To the best of our knowledge, no quantitative data on the ear movements in *R. rouxi* exist. However, [19] reported on the ear movements in the closely related bat *R. ferrumequinum*. Other authors found that *R. ferrumequinum* moves its pinnae back and forth in alternation over an angle of about 30 degrees [21]. The movement of one ear is in antiphase with respect to the other. This is, as one ear moves forward, the other one moves backwards. Based on this data, we simulate ear movements for rotations between −15 and 15 degrees in azimuth and elevation in steps of 5 degrees (0 degrees being the position in which the pinnae were as the animal was scanned). This is, we modeled the motion of the ears as a diagonal sweep from −15 degrees in azimuth and elevation to +15 degrees in azimuth and elevation. To test whether the results depend on the exact way the motion is modeled, we ran simulations in which the ears were either moved straight up-down (azimuth fixed to 0 degrees) or straight left-right (elevation fixed to 0). We found that the three motion patterns yielded very similar results.

Moving the pinnae was simulated by extracting a different part of the 360 degrees simulated sound field for each position. For each ear position we extracted an area of 180 degrees in elevation and in azimuth. It should be noted that, in order to simulate the pinnae movements, we rotated the HRTF but not the emission beam pattern. Therefore, the AHRTF is a combination of a rotated HRTF and a stationary emission beam pattern. Rotating the HRTF instead of rotating the ears with respect to the head is an approximation that is unlikely to influence the results. Indeed, we have previously shown that the influence of the head on the HRTF is small [6]. Moreover, we have run additional simulations to confirm this in *R. rouxi* (results not shown).

### Echolocation model

In this section of the paper, we outline our mathematical model (see [7] for a more detailed description) of the echolocation task. In each ear, we model the measured echo magnitudes in 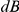 at 7 ear positions 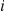 (−15 degrees to +15 degrees in steps of 5 degrees). The magnitudes received at the left and at the right ear are concatenated and stored in the vector 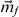 containing 14 elements. Note that this implies that modeling the pinnae to be moving in anti-phase only changes the order of the data in 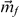. Therefore, under the current model, the phase relationship between the movement of the pinnae is of no importance for the outcome. Using the same measurement noise model as proposed in [7], the vector 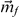 is assumed to be corrupted both by the unknown and varying reflector strength as well as the system noise. Their different effects on the vector 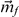 follow naturally if we represent the received echo magnitudes on a logarithmic scale (in 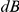), i.e., apply a compression very similar to the one performed by the hearing system. System noise is additive but, because of the logarithmic compression, its effect on 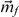 can be approximated by a maximum operator 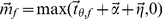
(2)

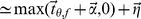
(3)with 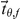 the template, i.e., the expected magnitude modulation at the different pinna positions (scaled such that 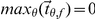), stored by the bat for reflector position 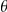 and frequency 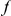. The noise level, i.e., the lower threshold below which no signal can be detected, is set at 0 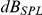. The vector 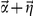 denotes the unknown and varying echo strength modulation due to the fluttering target. The term 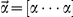 represents the mean echo strength averaged over the ear positions. As the noise level is set to zero the parameter 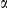 can be interpreted to specify the signal to noise ratio of the echo. The term 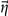 represents normally distributed multivariate noise, i.e. 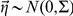 (the meaning of 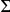 is explained in the next paragraph). This noise term models the unknown amplitude modulations imposed onto the echo due to target movement (e.g., fluttering target).

Following Bayes' theorem, the posterior probability 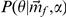 of a received vector 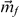 of strength 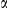 to originate from position 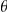 can be written as given by equation 4
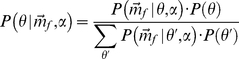
(4)


Taking into account that the expected value of 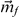, i.e., 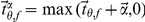, depends on 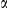, the likelihood of a received vector 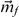 given a reflector position 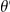 and echo strength 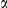 is calculated as,
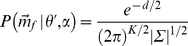
(5)with 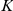 the total number of ear positions in the binaural template 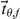 and
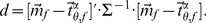
(6)


The covariance matrix 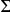 gives the variances and covariances of the stochastic vector 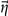. However, the magnitude of the echo 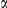 is unknown to the bat. Therefore, it is introduced as a nuisance parameter in the model,
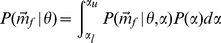
(7)with 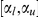 the range of 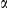 values that can occur. Hence, we rewrite equation 4 to arrive at,
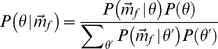
(8)


Equation 7 is calculated assuming that the bat considers all echo strengths in the interval 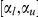 equally likely and thus maintains a uniform prior across reflector strengths. This is, we assume that the bat has no priori knowledge about the fraction of the impinging energy reflected by the target. Equation 8 gives the posterior distribution of 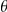. Using Shannon entropy, the uncertainty about the true target position when receiving a particular echo 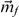 from position 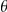 can be expressed in bits as,
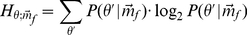
(9)


The quantity of direct behavioral relevance though is the average information 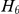 carried by all possible echoes 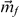 originating from position 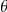. To calculate this quantity one should average over all realizations of the reflector ensemble. 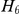 is approximated using a Monte Carlo simulation. For each frequency 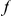 and position 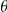, 20 realisations of the measurements 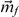 are generated. For each of these realizations, equations 4 to 9 are evaluated and the average value 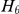 is reported. Twenty realizations for each frequency 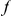 and position 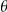 were found to yield stable results. Finally, in this paper, we mostly report on the global information transfer 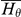 which averages 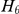 across the different target positions 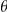.

The average entropy about the origin of an echo, can easilty be transformed in a measure of angular resolution as solid angle given by,
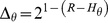
(10)with 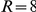 in our simulations.

### Estimation of the covariance matrix

**Figure 8:**
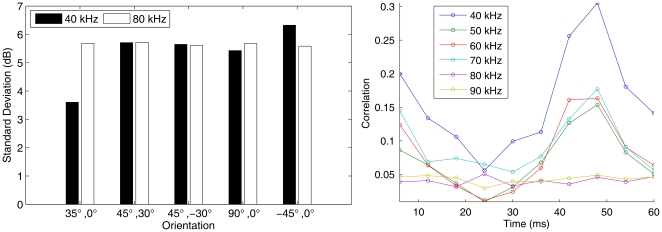
Results of measurements performed to estimate the parameter settings of the model. (a) The standard deviation of the spectral power of echoes from a fluttering locust at 40 and 80 kHz for five directions of ensonification. (b) The correlation between the magnitude of echoes from a fluttering locust as a function of the time between the collection of the echoes for 6 different frequencies (averaged across direction from which the insect was ensonified).

As outlined above, the model has only one free parameter, the covariance matrix 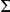. This matrix models the unknown amplitude modulations of the received echo due to target fluttering.
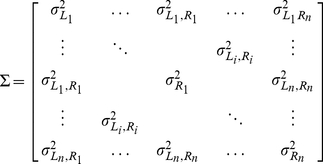
(11)with 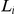 and 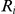 denoting the 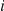-th position of the left and the right ear respectively. In our simulations, 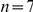. As can be seen from equation 11, three types of covariance values need to be filled in in this matrix. First, the variation for each of the positions of the two pinnae, 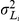 and 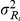. To obtain an estimate of this variance we ensonified a fluttering locust (*Locusta migratoria*, body length about 6 cm). The locust was attached in front of a Polaroid ultrasonic emitter. The distance between the locust and the emitter was 45 cm. The insect was ensonified using an hyperbolic FM sweep from 100 to 30 kHz, duration 1.5 ms. The fluttering locust was ensonified in batches of 400 calls with an interpulse interval of 6 ms yielding a repetition rate of about 166 Herz. The locust was ensonified from 5 different aspect angles to verify whether the estimation of 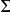 is aspect angle independent (see table 1). A Knowles microphone (Knowles Electronics, Itasca, IL, USA, FG23329) was mounted on top of the Polaroid emitter. For each angle, at least 20 batches of 400 measurements are collected, yielding a minimum of 8000 echoes. For each echo we extracted the spectral power at 40 and 80 kHz using the Goertzel algorithm. We calculated the standard deviation of the spectral power at these frequencies for each of the 5 positions from which the locust was ensonified. For the 5 positions and the 2 frequencies, the standard deviation of the gain was about 5 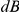. (figure 8). Therefore, we used 5 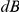 as the default value for the diagonal elements of 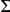. However, we also evaluated the model for 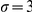 and 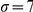. In the results presented above, we have labeled 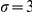, 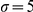 and 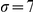 as Low, Medium and High noise respectively.

**Table 1:** The azimuth and elevation positions from which a fluttering locust was ensonified.

Directions	1	2	3	4	5
Azimuth	35	45	45	90	−45
Elevation	0	30	−30	0	0

The collected data also allowed us to estimate the value of 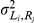,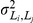 and 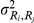 (the off-diagonal elements of 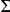). At 40 kHz, the correlation between the magnitude of any given measurement and one that is collected later varies between 0.1 and 0.3. For 80 kHz, the maximum correlation is less than 0.1 (figure 8). Based on this data, 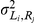,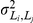 and 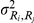 were set to 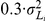. Other values were tested (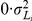, 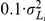 & 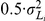) but were found to influence the results very little.

Finally, interaural covariances 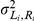 between a given measurement in one ear and one that is collected simultaneously in the other ear need to be determined. As the distance between the pinnae of *R. rouxi* is small compared to its hunting distance, this covariance was set to 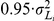 indicating a high correlation between the modulation due to target fluttering in the left and in the right ear. The values in 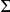 will depend on the species of prey ensonified by *R. rouxi*. Nevertheless, the presented measurements give plausible values for the different entries of 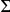.
